# Draft genome of the big-headed turtle *Platysternon megacephalum*

**DOI:** 10.1038/s41597-019-0067-9

**Published:** 2019-05-16

**Authors:** Dainan Cao, Meng Wang, Yan Ge, Shiping Gong

**Affiliations:** 1 Guangdong Key Laboratory of Animal Conservation and Resource Utilization, Guangdong Public Laboratory of Wild Animal Conservation and Utilization, Guangdong Institute of Applied Biological Resources, Guangzhou, 510260 China; 2 Novogene Bioinformatics Institute, Beijing, 100083 China

**Keywords:** Next-generation sequencing, Sequence annotation, Genome, Herpetology, DNA sequencing

## Abstract

The big-headed turtle, *Platysternon megacephalum*, as the sole member of the monotypic family Platysternidae, has a number of distinct characteristics including an extra-large head, long tail, flat carapace, and a preference for low water temperature environments. We performed whole genome sequencing, assembly, and gene annotation of an adult male big-headed turtle based on the Illumina HiSeq X genomic sequencing platform. We generated ~497.1 Gb of raw sequencing data (×208.9 depth) and produced a draft genome with a total length of 2.32 Gb and contig and scaffold N50 sizes of 41.8 kb and 7.22 Mb, respectively. We also identified 924 Mb (39.84%) of repetitive sequences, 25,995 protein-coding genes, and 19,177 non-coding RNAs. We generated the first *de novo* genome of the big-headed turtle; these data will be essential to the further understanding and exploration of the genomic innovations and molecular mechanisms contributing to its unique morphology and physiological features.

## Abstract (summary)

**Table:** 

Design Type(s)	sequence annotation objective • sequence assembly objective
Measurement Type(s)	whole genome sequencing assay
Technology Type(s)	next generation DNA sequencing
Factor Type(s)	
Sample Characteristic(s)	Platysternon megacephalum • stream

Machine-accessible metadata file describing the reported data (ISA-Tab format)

## Background & Summary

**Fig. 1:**
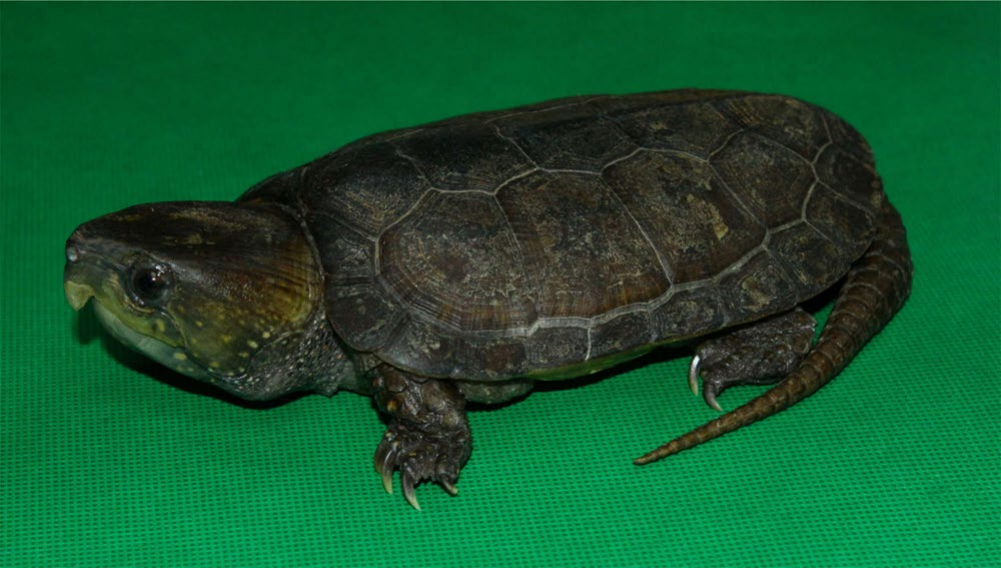
A representative big-headed turtle, *Platysternon megacephalum* in China.

The big-headed turtle, *Platysternon megacephalum* (NCBI Taxonomy ID: 55544), as the sole member of the family Platysternidae, is native to Southeast Asian countries, including China, Cambodia, Laos, Myanmar Thailand and Vietnam^1^. The species inhabits flowing cool rocky mountain streams with water temperatures usually between 12–28 °C^2^. It is an aquatic predator with strong climbing abilities, preying on lizard, frogs, fish, shrimps, crabs, snails, earthworms, insects, and even small birds and mammals, along with consuming some fruit and plant matter^3^. The big-headed turtle has some defining features, such as an extra-large head, long tail, and flat carapace. Its head width is approximately equal to half of its carapace width and therefore cannot be retracted into the shell. This species has the longest tail relative to body size of any turtle, with tail length being more than half of the carapace length^4^. In addition, it has a very flat carapace and an eagle-like hooked upper jaw (Fig. 1). The species prefers its body temperature to be around 25.3 °C^5^. While big-headed turtles are unique in many morphological and physiological characteristics compared to other turtle species, they are not unique in the anthropogenic threats they face.

The Asian turtle crisis has resulted in population declines in many species, including the big-headed turtle. Over harvesting for the pet trade, traditional medicine, and food represent the main drivers of the population decline and have resulted in an over 89% reduction in population density in South China^6^. The species was listed in the CITES (Convention on International Trade in Endangered Species of Wild Fauna and Flora) Appendix I in 2013 and as an endangered species in the IUCN (International Union for Conservation of Nature) Red List of Threatened Species in 2000^1^. Conservation and restoration measures are needed to protect this endangered species, and a genomic resource will be an essential tool for conservation efforts. However, there is currently little knowledge about the big-headed turtle’s genetic information, with previous research mainly focusing on mitochondrial DNA and the development of microsatellite markers^7–11^.

In this study, we report the first sequencing, assembly, and annotation of the big-headed turtle genome. The final draft genome assembly was approximately 2.32 Gb with a contig N50 of 41.8 kb and scaffold N50 of 7.22 Mb. A total of 25,995 protein-coding genes and 19,177 non-coding RNAs (409 rRNAs, 2,089 tRNAs, 16,050 miRNAs, and 629 snRNAs) were predicted from the genome assembly. The genomic resource of the big-headed turtle will be a key tool in the study of conservation genetics for this species.

## Methods

### Sample collection and sequencing

The genomic DNA of the big-headed turtle was extracted from the leg muscles of a single adult male obtained from Heyuan, Guangdong Province, China. The sampled turtle was one of a number that the law enforcement agencies confiscated from the black market and then transferred to scientific research institutions for study and captive breeding. Our institute has government permission to use confiscated big-headed turtles for scientific research (e.g. conservation genetics, ecology). The sampled turtle was euthanized and the animal collection and utility protocols were reviewed and approved by the Animal Ethics Committee at the Guangdong Institute of Applied Biological Resources (No: GIABR20170103). Ten paired-end libraries including four short-insert libraries (250 bp × 2, and 500 bp × 2) and six long-insert libraries (2 kb × 2, 5 kb × 2, and 10 kb × 2) were constructed and sequenced on an Illumina HiSeq X platform according to the manufacturer’s instructions (Illumina, San Diego, California, USA). The sequenced read length was 150 bp for each library. A total of 497.1 Gb (208.9×) of raw sequences were eventually obtained (Table 1). Prior to assembly, quality control was performed for raw reads using a SOAPfilter to filter out the adaptor sequences, the reads containing more than 10% unidentified nucleotides, and low-quality reads containing more than 50% bases with Illumina phred quality score ≤8. We obtained approximately 469.2 Gb of clean reads for further assembly.

**Table 1:** Statistics of big-headed turtle genome sequencing data.

Insert size	Libraries	Read length (bp)	Raw data		Clean data	
			Total data (Gb)	Sequence coverage (×)*	Total data (Gb)	Sequence coverage (×)*
250 bp	2	150	120.9	50.8	112.6	47.3
500 bp	2	150	103.6	43.5	96.5	40.5
2 Kbp	2	150	98.9	41.6	92.2	38.7
5 Kbp	2	150	78.6	33.0	75.5	31.7
10 Kbp	2	150	95.1	40.0	92.4	38.8
Total	10	—	497.1	208.9	469.2	197.1

*Sequence coverage was calculated based on the genome size of 2.38 Gb according to k-mer analysis.

### Assembly and evaluation

**Fig. 2:**
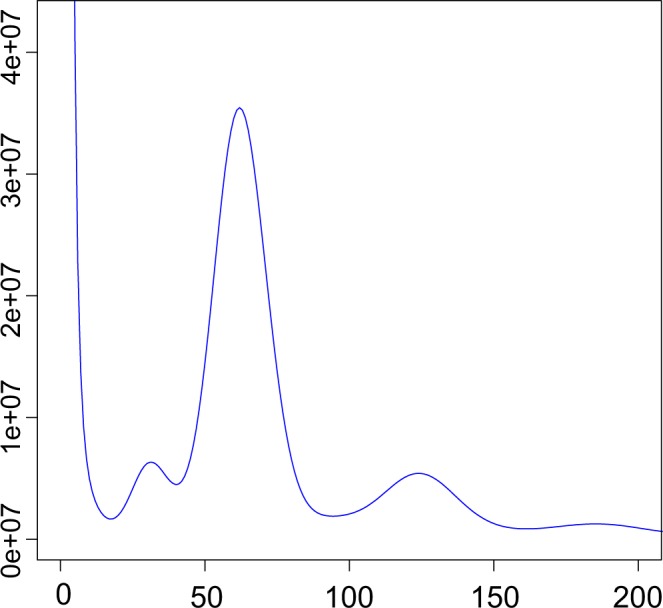
Distribution of 17-mer frequency. In total 170.2 Gb of high-quality short-insert reads (350 bp) were used to generate the 17-mer depth distribution curve frequency information.

A total of 170 Gb high-quality reads from the short-insert reads (350 bp) were used to estimate the genomic information of the big-headed turtle, and 17-mer frequency information was generated based on the K-mer analysis as implemented in the software GEC^12^. The heterozygous ratio was also evaluated based on the frequency of the heterozygous k-mers and homozygous k-mers using GCE software^12,13^. According to 17-mer analysis the estimated genome size of big-headed turtle was 2,383.87 Mb (~2.38 Gb), and the estimated heterozygous and repeat sequencing ratios were calculated to be 0.33 and 53%, respectively (Fig. 2 and Table 2).

**Table 2:** Estimation of the genome size using K-mer analysis.

Kmer	K-mer number	K-mer Depth	Estimated genome size (Mb)	Heterozygous Rate (%)	Repeat Rate (%)
17	134,817,220,976	56	2,383.87	0.33	53

*De novo* assembly was performed from the generated clean reads using SOAPdenovo^14^, a de Bruijn graph algorithm-based *de novo* genome assembler. We assembled the big-headed turtle genome in three steps: contig construction, scaffolding, and gap filling. First, three K (45, 59, 71) values were used to assemble the genome, according to the N50 length of contig and the BUSCO assessments of three genomes. The genome of 59-mer was chosen as the final genome for subsequent analysis. The clean reads of short-insert libraries (250 bp and 500 bp) were used to construct the contigs with 59-mer. Then reads of long-insert libraries (2 kb, 5 kb and 10 kb) were implemented to link the contig sequences into scaffold sequences. To further improve assembly quality, GAPcloser^14^ and SSPACE^15^ were applied to reduce gap regions and raise scaffold length using a genome sketch assembled by SOAPdenovo. This last step improved the contig N50 and N90 sizes to 41,757 and 5,528 bp, and the scaffold N50 and N90 sizes to 7,221,511 and 257,323 bp, respectively, with the fragments being longer than 100 bp (Table 3). The final assembly of the big-headed turtle genome had a total length of 2.32 Gb, which was similar to the three previously published turtle genomes: *Chrysemys picta bellii*^16^, *Chelonia mydas* and *Pelodiscus sinensis*^17^. The key assembly statistics of the big-headed turtle genome are comparable to or better than those of previously published turtle genomes (Table 4).

**Table 3:** Summary of the genome assembly.

Sample ID	Length		Number	
	Contig (bp)	Scaffold (bp)	Contig	Scaffold
Total	2,282,988,448	2,319,520,870	470,184	360,291
Max	453,655	40,162,337	—	—
Number >= 2000	—	—	100,848	13,027
N50*	41,757	7,221,511	15,524	84
N60	32,248	5,116,999	21,745	121
N70	23,516	3,324,563	30,018	177
N80	15,027	1,848,865	42,079	269
N90	5,528	257,323	65,609	576

*N50 referred to the scaffold larger than half the genome size which was added up from large to small.

**Table 4:** Summary statistics of four turtle genomes.

	*Chrysemys picta bellii*	*Chelonia mydas*	*Pelodiscus sinensis*	*Platysternon megacephalum*
Sequencing technology	Sanger + NGS	NGS	NGS	NGS
Assembly size (Gb)	2.59	2.24	2.21	2.32
Sequence coverage (×)	18.0	82.3	105.6	204.2
Contig N50 (kb)	11.9	20.4	21.9	41.8
Scaffold N50 (kb)	5,212	3,778	3,331	7,222
GC content (%)	43	43.5	44.4	44.63
Gene number	21,796	19,633	19,327	22,400

## Annotation

### Repeat annotation

There are two major types of repetitive sequences: tandem repeats and interspersed repeats^18^. For the repeat annotation of the big-headed turtle genome, both homology-based predictions and *de novo* methods were performed. In the homolog-based methods, interspersed repeats were identified using RepeatMasker^19^ and RepeatProteinMask to search against the published RepBase sequences. In the *de novo* method, RepeatMasker and RepeatModeler (http://www.repeatmasker.org/RepeatModeler.html) were used to detect interspersed repeats in the genome. Tandem Repeats Finder (TRF)^20^ was subsequently used to search for tandem repeats. Overall, the results identified a total of 924 Mb of non-redundant repetitive sequences in the big-headed turtle genome, which account for 39.84% of the whole genome (Table 5). The most predominant elements were long interspersed nuclear elements (LINEs), which accounted for 27.47% (637 Mb) of the genome (Table 6).

**Table 5:** Prediction of repeat elements in the big-headed turtle genome.

Type	Repeat Size (bp)	% of genome
Trf	47,338,094	2.04
Repeatmasker	874,588,835	37.71
Proteinmask	267,323,903	11.52
Total	924,094,854	39.84

**Table 6:** Statistics of repeat elements in the big-headed turtle genome.

Type	Length(bp)	% in Genome
DNA	125,220,062	5.40
LINE	637,155,697	27.47
SINE	9,369,097	0.40
LTR	217,629,821	9.38
Other	52	0.00
Satellite	1,062,050	0.05
Simple_repeat	990,682	0.04
Unknown	15,561,759	0.67
Total	903,544,627	38.95

### Structural annotation of genes

Three methods (homology-based, *ab initio* and transcriptome-based predictions) were used to predict gene structure in the big-headed turtle genome. In the homology-based method, the protein repertoires of *Alligator sinensis*, *Chelonia mydas*, *Chrysemys picta bellii*, *Deinagkistrodon acutus*, *Gallus gallus*, *Gekko japonicus*, *Nanorana parkeri*, *Parus major*, *Pelodiscus sinensis*, *Philomachus pugnax* and *Xenopus laevis* were downloaded from the NCBI database and mapped onto the big-headed turtle genome using TBLASTn^21^ with an E-value cutoff of 1 E^−5^. Then, homologous genome sequences were aligned against the matching proteins using GeneWise^22^ to define gene models. In the *ab initio* prediction, Augustus^23^, GlimmerHMM^24^ and SNAP^25^ were used to predict the coding regions of genes. To optimize the genome annotation, seven RNA tissue libraries (liver, skin, lung, intestine, heart, muscle and stomach) were constructed according to the manufacturer’s instructions (Illumina, San Diego, California, USA) and a total of 61.27 Gb of sequence data was generated. RNA-seq reads were used in *de novo* assembly with Trinity^26^. The unique transcriptional sequences were employed to predict gene models using PASA^27^. In total, 25,995 non-redundant protein-coding genes were annotated in the big-headed turtle genome (Table 7).

**Table 7:** Statistics of predicted genes.

Gene set		Number	Average transcript length (bp)	Average CDS length (bp)	Average exons per gene	Average exon length (bp)	Average intron length (bp)
Ab *initio* prediction	Augustus	29,895	20,126.36	1,083.78	5.51	196.7	4,222.41
	GlimmerHMM	163,252	12,105.64	451.4	3.91	115.37	4,001.41
	SNAP	83,162	36,326.76	549.05	3.61	152.28	1,3731.58
Homolog prediction	*Alligator sinensis*	57,621	9,467.09	794.29	3.45	230.02	3,535.42
	*Chelonia mydas*	37,629	15,048.25	1,093.96	4.9	223.12	3,575.20
	*Chrysemys picta bellii*	50,264	11,904.42	997.94	4.25	234.74	3,354.46
	*Deinag kistrodon acutus*	48,759	9,535.70	977.63	3.55	275.28	3,354.26
	*Gallus gallus*	45,081	11,902.30	950.46	3.91	243.04	3,762.56
	*Gekko japonicus*	34,511	14,745.27	1,136.85	4.8	236.7	3,578.43
	*Nanorana parkeri*	45,954	11,607.88	1,038.09	3.75	276.62	3,839.62
	*Parus major*	41,843	13,708.70	1,120.03	4.2	266.87	3,937.77
	*Pelodiscus sinensis*	46,338	11,270.86	981.39	4.05	242.04	3,368.40
	*Philomachus pugnax*	74,533	7,567.03	696.45	3.09	225.04	3,279.82
	*Xenopus leavis*	38,766	11,659.15	1,052.39	4.05	260	3,480.37
RNASeq	*PASA*	106,250	23,132.29	1,040.89	5.91	176.10	4,498.48
	*Cufflinks*	101,071	32,403.47	3,407.83	6.79	502.11	5,010.49
EVM		396,95	17,966.58	987.68	5.19	190.45	4,056.02
Pasa-update*		39,212	21,180.40	1,029.81	5.39	191.11	4,591.49
Final set*		25,995	30,713.64	1,298.80	7.33	177.28	4,649.67

*UTR regions were contained.

### Functional annotation of genes

The functional annotation of protein-coding genes of the big-headed turtle genome were predicted by aligning protein sequences against public databases including SwissProt (http://www.gpmaw.com/html/swiss-prot.html), KEGG (http://www.genome.jp/kegg/) and TrEMBL (http://www.uniprot.org) using BLASTp with an E-value cutoff of 1 E^−5^. Protein motifs and domains were annotated using InterPro^28^, and Gene Ontology (GO)^29^ terms for each gene were retrieved from the corresponding InterPro results. Overall, 22,400 protein-coding genes (86.2%) were successfully annotated (Table 8).

**Table 8:** Statistics of functional annotation.

Type	Number	Percentage (%)
Total	25,995	—
NR	22,357	86.0
Swiss-Prot	21,536	82.8
KEGG	19,560	75.2
InterPro	21,227	81.7
Pfam	19,277	74.2
GO	15,735	60.5
Annotated	22,400	86.2
Unannotated	3,595	13.8

### Non-coding RNA annotation

The non-coding RNAs were predicted in the big-head genome based on four categories: ribosomal RNA (rRNA), transfer RNA (tRNA), microRNAs (miRNA) and small nuclear RNA (snRNA). tRNAscan-SE^30^ was applied to identify tRNA with eukaryotic parameters according to the characteristics of tRNA. Because of the highly conserved characteristics of rRNA, BLASTN^31^ was used to predict rRNA sequences by aligning with a human template with an E-value of 1 E^−10^. The miRNA and snRNA sequences were identified using INFERNAL^32^ by searching against the Rfam database. We identified a total of 409 rRNA, 2,089 tRNA, 16,050 miRNA, and 629 snRNA genes in the big-headed turtle genome (Table 9).

**Table 9:** Summary of non-coding RNA.

Type		Number	Average length (bp)	Total length (bp)	% of genome
miRNA		16,050	85.58	1,373,585	0.05921
tRNA		2,089	74.90	156,473	0.00675
rRNA	rRNA	409	160.37	65,591	0.00283
	18S	77	164.01	12,629	0.00054
	28S	272	173.51	47,196	0.00204
	5.8S	4	113.50	454	0.00002
	5S	56	94.86	5,312	0.00023
snRNA	snRNA	629	129.16	81,241	0.00350
	CD-box	179	97.50	17,452	0.00075
	HACA-box	132	142.79	18,848	0.00081
	splicing	294	137.37	40,387	0.00174

## Data Records

This Whole Genome Shotgun project including the assembled genome sequence and the structural and functional annotation of genes has been deposited at DDBJ/ENA/GenBank under the accession QXTE00000000^33^. The version described in this paper is version QXTE01000000. Repeat annotation and Non-coding RNA annotation have been deposited at Figshare^34^. Raw read files have been deposited at NCBI Sequence Read Archive under the accession SRP156419^35^.

## Technical Validation

### Assessing the completeness of the genome assembly

To assess the quality of the genome assembly, we performed three independent evaluations as described below. First, the base content was counted with scaffolds longer than 100 bp and the results showed that the GC content for the big-headed turtle was 44.63% (Table 10), which was comparable to those of *Chrysemys picta bellii*, *Chelonia mydas* and *Pelodiscus sinensis* (Table 4). Second, the short-insert paired-end reads (250 bp and 500 bp) were mapped to the genome with BWA^36^. The mapping rate was 98.84% and the genome coverage was 99.57% (Table 11), indicating high reliability of genome assembly. Third, the SNPs (Single Nucleotide Polymorphisms) were counted to validate the uniformity of the genome using SAMtools^37^, and we found the ratio of homozygous SNPs was only 0.014% (Table 12), indicating that the assembly had a high base accuracy. Finally, CEGMA (http://korflab.ucdavis.edu/dataseda/cegma/) and BUSCO (http://busco.ezlab.org/) were used to evaluate the completeness of the assembly. CEGMA assessment showed that our assembly captured 226 (91.13%) of the 248 ultra-conserved core eukaryotic genes, of which 202 (81.45%) were complete (Table 13). BUSCO analysis showed that 95.2% and 2.6% of the expected vertebrate genes were identified as complete and fragmented, respectively, while 2.2% were considered missing in the assembly (Table 14).

**Table 10:** Base content statistics of the genome.

Type	Number (bp)	% of genome
A	632,158,059	27.25
T	631,959,166	27.25
C	509,623,076	21.97
G	509,248,147	21.97
N	36,532,422	1.57
Total	2,319,520,870	—
GC*	1,018,871,223	44.63

*GC content of the genome without N.

**Table 11:** Statistics of mapping ratio in genome.

Type	Content	Value
Reads	Mapping rate (%)	98.84
Genome	Average sequencing depth (×)	72.05
	Coverage (%)	99.57
	Coverage at least 4× (%)	98.73
	Coverage at least 10× (%)	97.25
	Coverage at least 20× (%)	95.38

**Table 12:** Number and density of SNPs in big-headed turtle genome.

Type	Number	Proportion (%)
All SNPs	5,319,363	0.233%
Heterozygous SNPs	4,999,745	0.219%
Homozygous SNPs	319,618	0.014%

**Table 13:** Assessment of CEGMA.

Species	Complete		Complete + Partial	
	Proteins	Completeness (%)	Proteins	Completeness (%)
Big-headed turtle	202	81.45	226	91.13

**Table 14:** Assessment of BUSCO.

Species	Size	BUSCO notation assessment results*
Big-headed turtle	2320 Mb	C: 95.2% [S: 94.2%, D: 1.0%], F: 2.6%, M: 2.2%, n: 2586

*C: Complete BUSCOs; S: Complete and single-copy BUSCOs; D: Complete and duplicated BUSCOs; F: Fragmented BUSCOs; M: Missing BUSCOs; n: Total BUSCO groups searched.

### Annotation filtering and validation

The EVM software^38^ was used to merge the above results of gene annotation and 39,212 genes were obtained from the merged set. To further revise the genome annotation, we removed the following type of genes: (1) genes with overlap regions of TE ≥ 20%; (2) premature termination genes; (3) genes with only *de novo* predictive support. After filtering, 25,995 genes were retained. In addition, total RNA-seq reads of 7 tissues were mapped onto the big-headed turtle genome to further identify exon regions and splice positions using Tophat^39^ and Cufflinks^40^, and 20,028 (77.05%) genes had evidence supports of RNA data (RPKM value > 1) cross above 7 tissues.

## ISA-Tab metadata file

Download metadata file

## Data Availability

The execution of this work involved using many software tools, whose settings and parameters are described below. (**1**) **GCE**: version1.0.0, parameters: -H 1; (**2**) **SOAPdenovo**: version2, k-mer size of 59; (**3**) **GAPcloser**: version1.12, parameters: -l 150 -p 31; (**4**) **SSPACE**: version3.0, default parameters; (**5**) **RepeatMasker**: Repeat Masker-open-4-0-6, parameters: -a -nolow -no_is -norna -parallel 1; (**6**) **RepeatModeler**: RepeatModeler-open-1.0.11, parameters: -database genome -engine ncbi -pa 15; (**7**) **Tandem Repeats Finder**: TRF-407b, parameters: 2 7 7 80 10 50 2000 -d -h; (**8**) **TBLASTn**: blast-2.2.26, parameters: -p tblastn -e 1e-05 -F T -m 8 -d; (**9**) **GeneWise**: version2.4.1, parameters: -tfor -genesf -gff; (**10**) **Augustus**: version3.2.3, parameters: –uniqueGeneId = true–noInFrameStop = true–gff3 = on–genemodel = complete–strand = both; (**11**) **GlimmerHMM**: version3.0.1, parameters: -g -f; (**12**) **SNAP**: snap-2013-11-29, default parameters; (**13**) **Trinity**: trinityrnaseq-2.1.1, parameters: –seqType fq-CPU 20–max_memory 200G–normalize_reads–full_cleanup–min_glue 2–min_kmer_cov 2–KMER_SIZE 25; (**14**) **PASA**: PASA_r20140417, default parameters; (**15**) **InterPro**: version29.0, perl-based version4.8, default parameters; (**16**) **tRNAscan-SE**: tRNAscan-SE-1.3.1, default parameters; (**17**) **INFERNAL**: version1.1rc4 (June 2013); (**18**) **BLASTp**: blast-2.2.26, parameters: -p blastn -e 1e-10 -v 10000 -b 10000; (**19**) **BWA**: bwa-0.7.8, parameters: mem -k 32 -w 10 -B 3 -O 11 -E 4 -t 10; (**20**) **SAMtools**: samtools-0.1.19, parameters: mpileup mpileup -m 2 -u; (**21**) **EVM**: VidenceModeler-1.1.1, parameters: –segmentSize 200000–overlapSize 20000; (**22**) **Tophat**: tophat-2.0.13, parameters: -p 6–max-intron-length 500000 -m 2–library-type fr-unstranded; (**23**) **Cufflinks**: cufflinks-2.1.1, parameters: -I 500000 -p 1–library-type fr-unstranded -L CUFF; (**24**) **BUSCO**: version3.0.2, OrthoDBv9_vertebrata.
